# SQSTM1/p62 and Hepatic Mallory-Denk Body Formation in Alcohol-Associated Liver Disease

**DOI:** 10.1016/j.ajpath.2023.02.015

**Published:** 2023-10

**Authors:** Hui Qian, Wen-Xing Ding

**Affiliations:** ∗ Department of Pharmacology, Toxicology, and Therapeutics, The University of Kansas Medical Center, Kansas City, Kansas; † Department of Internal Medicine, The University of Kansas Medical Center, Kansas City, Kansas

## Abstract

Sequestosome 1 (SQSTM1/p62; hereafter p62) is an autophagy receptor protein for selective autophagy primarily due to its direct interaction with the microtubule light chain 3 protein that specifically localizes on autophagosome membranes. As a result, impaired autophagy leads to the accumulation of p62. p62 is also a common component of many human liver disease–related cellular inclusion bodies, such as Mallory-Denk bodies, intracytoplasmic hyaline bodies, α_1_-antitrypsin aggregates, as well as p62 bodies and condensates. p62 also acts as an intracellular signaling hub, and it involves multiple signaling pathways, including nuclear factor erythroid 2–related factor 2, NF-κB, and the mechanistic target of rapamycin, which are critical for oxidative stress, inflammation, cell survival, metabolism, and liver tumorigenesis. This review discusses the recent insights of p62 in protein quality control, including the role of p62 in the formation and degradation of p62 stress granules and protein aggregates as well as regulation of multiple signaling pathways in the pathogenesis of alcohol-associated liver disease.

Long-term alcohol consumption can lead to alcohol-associated liver disease (ALD), which is the leading cause of cirrhosis-related deaths.^1^ In line with the *Global Status Report on Alcohol and Health* in 2018 from World Health Organization, alcohol-attributable liver cirrhosis caused >600,000 deaths.^2^ The pathogenesis of ALD includes alcoholic fatty liver, alcoholic hepatitis (AH), fibrosis, cirrhosis, and hepatocellular carcinoma (HCC).^2^, ^3^, ^4^ According to the spectrum studies of ALD, approximately 90% to 100% heavy alcohol drinkers develop fatty liver, which is manifested as increased accumulation of lipid droplets (LDs) in hepatocytes. Nearly one of four drinkers with fatty liver develop AH, which is characterized by increased hepatocyte death and hepatic inflammation. Up to one of five patients with AH advanced to cirrhosis, with the liver becoming irreversibly scarred, and finally a few heavy drinkers can even develop HCC.^5^^,^^6^ A recent report indicates that the number of patients with ALD was significantly increased during the COVID-19 pandemic in the United States.^7^ Moreover, alcohol consumption synergistically promotes the progression of viral hepatitis.^8^^,^^9^ In a modeling study, Julien et al predicted that ALD will increase 84% in 2040 compared with 2019 under the current drinking rates.^10^ Notably, alcohol-related liver cirrhosis is becoming a leading cause of elevated mortality and morbidity worldwide.^11^^,^^12^ As the aging population increases and becomes a new global issue,^13^^,^^14^ the prevalence of ALD in the elderly population is also increasing.^15^, ^16^, ^17^ A wide variety of factors associated with aging, such as increased hepatic infiltration of inflammatory cells,^18^ decreased alcohol metabolism,^19^ and reduced liver regeneration,^20^ can aggravate ALD in elderly people. Both ALD and aging are associated with dysregulated autophagy, and compromised autophagy is a hallmark of aging.^21^, ^22^, ^23^ Despite remarkable progress in understanding the pathogenesis of ALD, alcohol abstinence is still the most efficient treatment for all stages of ALD, and liver transplantation is the only effective treatment for end-stage ALD.^24^^,^^25^ Thus, there is an urgent need to better understand the pathologic mechanisms of ALD, which may help to identify potential therapeutic targets for treating ALD.

## Autophagy and Alcohol-Induced Liver Injury

Autophagy is an evolutionarily conserved degradation process for removing protein aggregates or damaged organelles. Autophagy can be classified into three major types, chaperone-mediated autophagy (CMA), microautophagy, and macroautophagy, according to how cargos that contain disposable materials are delivered to lysosomes.^4^^,^^26^ The detailed process of these three types of autophagy and the function of autophagy-related genes have been well described in previous reviews.^4^^,^^26^^,^^27^ Studies on the role of microautophagy in liver biology and diseases are scarce likely because of the lack of specific tools to separate microautophagy from autophagy and CMA. CMA degrades a subset of proteins that consist of the KFERQ motif using the lysosome-associated membrane protein type 2A as the receptor.^28^ CMA declines in aged mouse livers, and loss of hepatic CMA in mice leads to increased hepatic oxidative stress, decreased ability of drug metabolism, and proteostasis, resulting in liver dysfunction.^29^ Future studies are needed to investigate the role and relevance of microautophagy and CMA in ALD. Macroautophagy (hereafter referred to as autophagy) initiates from the nucleation of phagophores (also called isolated membrane) and follows by the expansion of phagophores into double membrane autophagosome. Autophagosome and its engulfed cytoplasm materials and damaged organelles then fuse with lysosomes to form autolysosome, resulting in degradation of the engulfed contents.^4^ Accumulated evidence indicates that autophagy has emerged as a key player in liver physiology, and it helps maintain liver homeostasis, balance liver metabolism, and liver regeneration.^30^, ^31^, ^32^, ^33^ In line with this notion, deficiency of autophagy has been associated with multiple liver diseases, especially in ALD^34^, ^35^, ^36^ and HCC.^37^, ^38^, ^39^ Deletion of autophagy-related 7 (*Atg7*) or *Atg5* using either albumin-Cre or adeno-associated virus encoding Cre-recombinase under the control of hepatocyte-specific thyroxine-binding globulin promoter (AAV-TBG-Cre) leads to severe liver injury, inflammation, and spontaneous liver tumors with persistent nuclear factor erythroid 2–related factor 2 (NRF2) activation.^38^, ^39^, ^40^, ^41^, ^42^ Interestingly, using an inducible Ubc-cre to delete whole-body *Atg7* in mice only leads to mild liver pathologic changes with no persistent NRF2 activation.^43^ However, these differences are likely due to insufficient deletion of hepatic *Atg7* by Ubc-cre in this inducible whole-body *Atg7* knockout mice.

Prior studies suggest that autophagy has an important role in protecting against acute alcohol-induced liver injury by the clearance of damaged mitochondria and hepatic LDs,^44^ whereas long-term alcohol consumption impairs autophagy and aggravates ALD.^36^^,^^45^, ^46^, ^47^ Alcohol directly impairs autophagy activity by downregulating hepatic expression of transcription factor EB (TFEB) via activation of mechanistic target of rapamycin complex 1 (mTORC1), which reduces lysosomal biogenesis and exacerbates alcohol-induced liver injury in mice.^45^ Overexpression of TFEB increases the expression of autophagy-related and lysosomal genes and protects against alcohol-induced steatosis and inflammation in mice.^45^ Alcohol-fed mice have impaired or insufficient lipophagy, a selective autophagy for removing excess LDs, causing lipid accumulation and liver steatosis.^34^^,^^48^ Mechanistically, alcohol decreases hepatic RAB7 activity, resulting in decreased fusion of autophagosome with LDs.^49^ Alcohol also decreases levels of phosphorylated Src kinase and dynamin 2, causing decreased lysosomes and lysosome reformation and impaired LD breakdown.^48^^,^^50^ In addition, ethanol treatment elevates ubiquitin signals on LDs, which further recruites autophagy adaptor protein p62 to the LDs to promote lipophagy in AML12 cells.^51^ Moreover, enhanced lipophagy by quercetin ameliorates ethanol-induced liver steatosis.^52^ Although these findings on lipophagy in cultured cells are interesting, mice with genetic deletion of autophagy-related genes [ie, *Atg5, Atg7,* or *Fip200* (alias *Rb1cc1*)] do not develop hepatic steatosis in response to physiologic starvation or partial hepatectomy or high-fat diet feeding.^53^, ^54^, ^55^, ^56^ Decreased steatosis in autophagy-deficient livers occurs partially due to the accumulation of nuclear receptor co-repressor 1, which inhibits liver X receptor α–mediated *de novo* lipogenesis and LD biogenesis.^54^ Notably autophagy-deficient livers have remarkably high levels of p62 that leads to NRF2 activation, which may also contribute to the resistance to starvation-induced hepatic steatosis.^53^ Therefore, the relevance of lipophagy *in vivo* needs to be further investigated.

In addition to lipophagy, mitophagy, a selective autophagy that specifically targets damaged mitochondria, plays a protective role in alcohol-induced liver injury by eliminating damaged mitochondria that otherwise can elevate intracellular reactive oxygen species and cause cell damage.^57^, ^58^, ^59^, ^60^ Alcohol consumption blunts mitophagy through reducing the mitochondrial fission protein dynamin-related protein 1 in mouse liver and augmented production of megamitochondria, a hallmark of ALD. Loss of hepatic dynamin-related protein 1 increases metabolic stress through mitochondrial maladaptation likely due to impaired mitophagy because megamitochondria are difficult to remove by mitophagy because of their size.^61^ Both short-term and long-term ethanol exposure can induce Parkin mitochondrial translocation and increase Parkin-mediated mitophagy, which serves as another layer of adaptive protection against alcohol-induced liver injury.^62^^,^^63^

Overall, current data support a temporal role of autophagy in ALD and impaired autophagy, including dysfunction of mitophagy, lipophagy, and lysosome activity, in long-term alcohol exposure, resulting in aggravated ALD. Short-term alcohol exposure induces adaptive protective autophagy, whereas this adaptive autophagy process becomes impaired and lost in long-term alcohol exposure.^36^ Pharmacologic intervention of various types of autophagy may serve as new therapeutic avenues for ALD, such as balancing hepatic metabolism (mitophagy), increasing lipid degradation (lipophagy), and promoting clearance of hepatic protein aggregates.

One of the hallmarks of ALD is the accumulation of hepatic Mallory-Denk bodies (MDBs), which are hepatic inclusion bodes and protein aggregates.^64^ Sequestosome 1 (SQSTM1/p62, hereafter p62) is a main component of MDBs, which is also a substrate of autophagy that accumulates in autophagy-deficient mouse livers.^65^, ^66^, ^67^ The basic structure of p62, p62-mediated signaling pathways, and selective autophagy as well as their contributions to the pathogenesis of ALD is discussed below.

## Domain Structure of p62

p62 is a multidomain protein that interacts with various proteins via its different domains and acts as an intracellular signaling hub for many different pathways.^68^, ^69^, ^70^ The N-terminal Phox and Bem1 (PB1) domain of p62 leads to the formation of p62 homo-oligomerization.^71^ The PB1 domain of p62 also interacts with other PB1-containing proteins, such as atypical protein kinase Cζ (PKCζ), to form hetero-oligomers,^71^ and interacting with the neighbor of *BRCA1* gene 1 (NBR1), an autophagy receptor with a domain architecture similar to that of p62.^72^ Mitogen-activated protein kinase kinase kinase 3 (MEKK3) also contains a PB1 domain, which forms a heterodimer with the PB1 domain of p62 and binds to tumor necrosis factor receptor–associated factor 6 (TRAF6), a lysine 63 E3 ligase, to trigger NF-κB activation.^73^ The PB1 domain is followed by a ZZ-type zinc finger domain, which is required for efficient starvation-induced autophagy in mouse embryonic fibroblasts.^74^ Moreover, the ZZ domain binds to the receptor interacting protein to regulate NF-κB activation.^70^ The TB domain next to the ZZ domain also activates NF-κB via interacting with TRAF6.^70^ Additionally, p62 interacts with the regulatory-associated protein of mTOR via the region between the ZZ and TB domains to activate mTORC1.^75^ p62 directly binds to microtuble light chain 3 (LC3) through the LC3-interacting region (LIR) and thus acts as an autophagy receptor protein for selective autophagy.^76^^,^^77^ Followed by the LIR domain is a Kelch-like ECH-associated protein 1 (KEAP1) interacting region (KIR) that binds to KEAP1 and drives KEAP1 degradation by selective autophagy, resulting in NRF2 activation via the noncanonical KEAP1-NRF2 pathway.^66^^,^^78^, ^79^, ^80^ p62- and KEAP1-positive aggregates have been observed in the autophagy-deficient mouse livers, causing the persistent activation of NRF2 in the liver.^39^^,^^67^^,^^81^ The ubiquitin-associated (UBA) domain on C-terminal binds to ubiquitin-labeled proteins or damaged organelles and leads them into autophagosome for degradation.^70^^,^^82^

**Figure 1:**
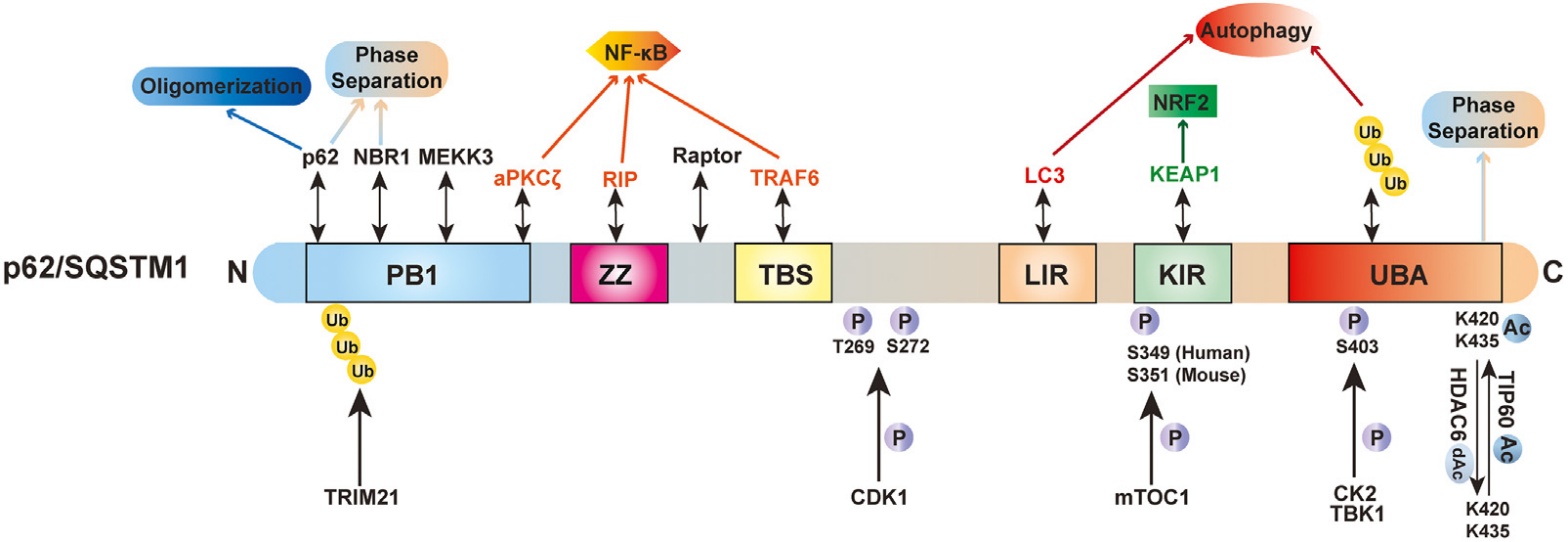
Schematic domain structure of sequestosome 1 (SQSTM1/p62). The Phox and Bem1 (PB1) domain of p62, interacting with PB1-containing proteins, such as p62, neighbor of the *BRCA1* gene 1 (NBR1), atypical protein kinase Cζ (aPKCζ), or mitogen-activated protein kinase kinase kinase 3 (MEKK3) to form homo-oligomers or hetero-oligomers. The ubiquitination of the PB1 domain is mediated by tripartite motif 21 (TRIM21) to inhibit the oligomerization. The ZZ-type zinc finger (ZZ) interacts with the receptor interacting protein (RIP), the tumor necrosis factor receptor–associated factor 6 (TRAF6)–binding domain (TBS) interacts with TRAF6, and PKCζ activates the p62-mediated NF-κB pathway. p62 binds to the microtubule light chain 3 (LC3) protein through the LC3-interacting region (LIR) domain to trigger selective autophagy. p62 activates the noncanonical Kelch-like ECH-associated protein 1 (KEAP1)–nuclear factor erythroid 2–related factor 2 (NRF2) pathway by interacting with KEAP1 for its degradation via the KEAP1 interacting region (KIR) domain. The ubiquitin (UB)–associated (UBA) domain on the C terminus binds to ubiquitinated proteins. Phosphorylation of Ser403 residue on the UBA domain and Ser349 residue on KIR occur in response to selective autophagy. Both the PB1 and UBA domains play an important role in p62-mediated phase separation, which is enhanced by acetylation at K420 and K435 of p62. Ac, acetylation; C, C-terminal; CDK1, cyclin-dependent kinase 1; HDAC6, deacetylase histone deacetylase 6; N, N-terminal; P, phosphorylation; Raptor, regulatory-associated protein of mechanistic target of rapamycin; TBK1, TANK-binding kinase 1.

Although p62 can interact with LC3, KEAP1, and ubiquitinated proteins, these interactions are relatively weak. However, these weak interactions can be enhanced by the oligomerization and phosphorylation of p62 on various sites. PB1 domain–mediated oligomerization of p62 increases the binding affinities between p62 and other proteins, including the UBA domain of p62 with ubiquitinated misfolded proteins and the LIR domain of p62 with LC3. This promotes the relocation of ubiquitinated protein aggregates to autophagosomes for degradation.^83^ Casein kinase 2 directly phosphorylates Ser403 of p62 to increase the autophagic clearance of ubiquitinated proteins and protein aggregates.^84^ Additionally, polyubiquitinated mitochondria recruites TANK-binding kinase 1 (TBK1) through TBK1-binding adaptor proteins, including optineurin (OPTN).^85^ TBK1 is activated by autophosphorylation and phosphorylates p62 at S403, which further strengthens binding between the UBA domain and ubiquitin that drives the ubiquitinated mitochondria into autophagosome for degradation.^85^ mTORC1 phosphorylates p62 at Ser349 (humans) or Ser351 (mice) to enhance the binding affinity between the KIR domain of p62 and KEAP1, resulting in constant NRF2 activation through the noncanonical KEAP-NRF2 pathway.^86^ Both the tripartite motif (TRIM) 16 and TRIM21 proteins have E3 ligase activities, and they ubiquitylate p62 under oxidative and proteotoxic stress conditions.^87^^,^^88^ However, these two proteins play different roles during stress. TRIM16 acts as a scaffold protein to interact with p62, KEAP1, and ubiquitinated proteins, leading to the degradation of protein aggregates and stabilizing NRF2 activation against oxidative stress.^87^ In contrast to TRIM16, TRIM21 prevents p62 oligomerization and releases the p62 sequestrated KEAP1 by ubiquitylating PB1 domain of p62 at lysine (K)7 via lysine 63 linkage, leading to a decrease in p62-mediated autophagy activity and an increase in KEAP1-mediated NRF2 degradation to aggravate oxidative stress and liver carcinogenesis.^88^^,^^89^ In addition, cyclin-dependent kinase 1 phosphorylated p62 at Thr269 and Ser272 during mitosis, regulating cell cycle progression, cell proliferation, and tumorigenesis.^70^ Because of the unique protein structure of p62 and its capacity to interact with multiple proteins in various signaling pathways, it is not surprising that p62 has critical roles in regulating redox, proteostasis, cell death, cell survival, proliferation, and tumorigenesis.^70^^,^^90^^,^^91^ In addition to phosphorylation and ubiquitination, p62 can also be acetylated by acetyltransferase TIP60 and deacetylated by deacetylase histone deacetylase 6. Acetylation at K420 and K435 increases the binding of p62 to ubiquitin and facilitates polyubiquitin chain–induced p62 phase separation by disrupting UBA dimerization.^92^ In cells under stress, increased p62 acetylation may thus promote selective degradation of ubiquitinated proteins by regulating the assembly of p62 bodies.^92^ The schematic domain structure and posttranslational modifications of p62 is illustrated in Figure 1 and its interacting partners in Table 1.^93^, ^94^, ^95^, ^96^, ^97^, ^98^

**Table 1:** Function of Interaction between p62 and Its Partners or Postmodifications

Domain of p62	Partners or postmodification	Functions
Phox and Bem1 (PB1) domain	PB1-containing protein: p62 (sequestosome-1)	PB1 domains of p62 forms homo-oligomerization.^71^
	Atypical protein kinase Cζ (aPKCζ)	PB1 domains of p62 and aPKCζ form hetero-oligomers.^71^
	Neighbor of *BRCA1* gene 1 (NBR1)	PB1 domains of p62 and NBR1 form oligomer.^72^^,^^93^
	Mitogen-activated protein kinase kinase kinase 3 (MEKK3)	PB1 domains of p62 and MEKK3 form heterodimer, binding to TRAF6, a lysine 63 (K63) E3 ligase, to trigger NF-κB activation.^73^
	TRIM21 (tripartite motif)	TRIM21 ubiquitylates PB1 domain of p62 under oxidative and proteotoxic stress conditions to prevent p62 oligomerization.^87^, ^88^, ^89^
ZZ-type zinc finger (Znf) domain	Receptor interacting protein (RIP)	The interaction between p62 and RIP regulates NF-κB activation.^70^ p62 interacts with RIP and links RIP to aPKC (specific two isoforms PKCζ and PKCλ), which are involved in NF-κB activation.^94^
TB domain	Tumor necrosis receptor–associated factor 6 (TRAF6)	The TB domain of p62 interacts with TRAF6 to activate NF-κB in response to IL-1 stimulation.^70^^,^^95^ p62 also involves in other TRAF6-dependent signaling pathways to activate NF-κB in response to other inducers, such as CD40, receptor activator of NF-κB ligand, and nerve growth factor.^96^, ^97^, ^98^
The region between the ZZ and TB domains	Regulatory-associated protein of mechanistic target of rapamycin (mTOR) (Raptor)	p62 interacts with Raptor via the region between the ZZ and TB domains to activate mTOR complex 1 (mTORC1).^75^ Moreover, p62-Raptor interaction favors mTORC1 in the presence of S6 kinase β1.^75^
Sites: Thr269 and Ser272	Cyclin-dependent kinase 1 (CDK1)	CDK1 phosphorylates p62 at Thr269 and Ser272 to regulate cell cycle progression, cell proliferation, and tumorigenesis.^70^
LC3-interacting region (LIR)	Microtubule-associated protein 1 light chain 3 (LC3)	p62 directly binds to LC3 through the LC3-interacting region (LIR) and thus acts as an autophagy receptor protein for selective autophagy.^76^^,^^77^
KEAP1-interacting region (KIR)	Kelch-like ECH-associated protein 1 (KEAP1)	KIR binds to KEAP1 and drives KEAP1 degradation via selective autophagy, resulting in NRF2 activation via the noncanonical KEAP1-NRF2 pathway.^66^^,^^78^, ^79^, ^80^ p62 and KEAP1-positive aggregates have been observed in the autophagy-deficient mouse livers, causing the persistent activation of NRF2 in the liver.^39^^,^^67^^,^^81^
	Mammalian target of rapamycin complex 1 (mTORC1)	mTORC1 phosphorylates p62 at Ser349 (humans) or Ser351 (mice) to enhance the binding affinity between KIR domain of p62 and KEAP1 resulting in persistent NRF2 activation.^86^
Ubiquitin-associated domain (UBA)	Casein kinase 2 (CK2)	CK2 phosphorylates Ser403 of p62 to increase the autophagic clearance of ubiquitinated proteins and protein aggregates.^84^
	TANK-binding kinase 1 (TBK1)	TBK1 is activated by autophosphorylation and phosphorylates p62 at S403, which further strengthens binding between the UBA domain and ubiquitin.^85^
	Acetyltransferase (TIP60)	Acetylation at K420 and K435 increases the binding of p62 to ubiquitin by disrupting UBA dimerization and facilitates polyubiquitin chain–induced p62 phase separation.^92^
	Deacetylase histone deacetylase 6 (HDAC6)	HDAC6 direct interacts with p62 and deacetylates p62 at K420 and K435.^92^

## Role and Mechanism of p62 in Regulating Protein Aggregates and Condensates on Long-Term Alcohol Consumption

Because of its unique multidomain structure and interactions with various proteins, p62 not only plays a role in selective autophagy but also contributes to the formation of stress granules (SGs) and ubiquitin-positive protein aggregates in the cytoplasm.^3^ p62 is a common component of many human disease–related cellular inclusion bodies, such as MDBs, intracellular hyaline bodies (IHBs), and α_1_-antitrypsin aggregates in the liver^99^, ^100^, ^101^ as well as Lewy bodies, neurofibrillary tangles, and huntingtin aggregates in the brain.^70^^,^^101^ Notably, all these inclusion bodies are also positive for ubiquitin.^101^ Unlike soluble proteins that can be removed by ubiquitin proteasome system, insoluble protein aggregates can only be removed by autophagy.^102^^,^^103^ Therefore, the presence of hepatic protein aggregates may act as a sign for decreased autophagy activity.

## Role of p62 in MDBs and IHBs

MDBs, which are membrane-less cytoplasmic protein aggregates, are found in three-fourths of patients with AH and in nearly all patients with alcoholic cirrhosis.^104^ MDBs were first discovered in a patient with AH by professor Frank B. Mallory in 1911.^64^ They have been found in ballooned hepatocytes and are associated with ALD.^105^ The existence of MDBs in hepatocytes is a hallmark of ALD, although whether and how MDBs contribute to ALD remains elusive.^105^^,^^106^ Multiple factors contribute to MDB formation. Mice exposed to 3,5-diethoxycarbonyl-1,4-dihydrocollidine and high-fat diet show increased MDB formation and develop severe liver injury and inflammation.^64^^,^^107^ Sex also plays a crucial role in MDB formation, with one study showing that male mice formed significantly more MDBs than female mice, which is likely due to higher levels of estradiol and lower levels of oxidative stress in female mice.^108^ In addition, aging mice are more susceptible to MDB formation compared with young mice due to increased oxidative stress and decreased autophagy and proteasome activities.^109^ Animal models are essential to investigate the pathogenesis and mechanisms of ALD. Unfortunately, current ALD animal models do not faithfully phenocopy the full spectrum of human ALD because these animal models fail to recapitulate the characteristics of severe human ALD, such as fibrosis, ductular reaction, and accumulation of MDBs and hepatic progenitor cells.^110^ One potential approach is to combine a 3,5-diethoxycarbonyl-1,4-dihydrocollidine diet and alcohol feeding in mice, which increases cholestasis and hepatic progenitor cells, although MDBs were not investigated in this model.^111^

The major components of MDBs are keratin 8/18, ubiquitinated proteins, chaperone proteins, misfolded proteins, transglutaminase-2, and p62. The indispensable step of MDB formation is stress-induced up-regulation of K8, which is cross-linked by transglutaminase-2.^112^^,^^113^ The increased ubiquitinated K8 and ubiquitinated misfolded proteins overwhelm proteasome and other protein quality control machineries, resulting in the accumulation of MDBs.^114^ The UBA domain of p62 directly binds to ubiquitinated K8 and ubiquitinated misfolded proteins, which further sequesters them into aggresome or MDBs via the PB1 domain of p62.^115^ p62 appears to play a critical role in MDB formation because p62 knockout mice manifest defective MDB maturation and fail to form large MDBs, although this does not affect modifications of keratin.^109^ Some studies indicate that p62 reduces cytotoxicity by sequestering soluble misfolded protein into insoluble and less toxic aggresomes or sequestosome-like aggregates, such as MDBs.^70^ In addition to regulating MDB formation, as a substrate of selective autophagy, p62 also mediates MDB degradation by autophagy.^64^ Therefore, the balance between the formation and degradation mediated by p62 may determine the levels of MDBs in ALD.

Although the reticular MDBs have multiple components, the globular intracytoplasmic hyaline bodies only consist of p62 and ubiquitin or only p62.^116^ A previous study showed that expression of p62 alone leads to the formation of the p62-containing aggregates IHBs; however, presence of abnormal keratins and p62 at the same time results in the formation of MDBs.^99^ Both MDBs and IHBs have been detected in HCC, in which MDBs occur in approximately 20% to 30% of HCCs, whereas IHBs are seen in approximately 20% of the cases.^99^^,^^100^^,^^117^ In addition, HCCs with IHB show worse prognosis than HCCs without IHBs, but the function of IHBs is still largely unclear.^116^ IHBs are also eliminated by p62-mediated autophagy, and an increase in p62-containing protein aggregates is regarded as a marker of impaired autophagy.^64^^,^^118^ Whether MDBs and IHBs would also contain KEAP1 and regulate noncanonic NRF2 activation remains unclear. Future studies are needed to further investigate whether and how p62-positive IHBs or MDBs contribute to the liver injury and tumorigenesis in autophagy-deficient livers and ALD.

## Role of p62 in SGs

During the past few years, the presence of a number of non–membrane-bound body or organelles inside the cell, such as SGs, germ granules, Cajal body, and nucleolus, has drawn a lot of research attention and expanded our current knowledge on their cellular organization and functions.^119^ SGs can rapidly form in cells during stress as a mechanism to combat the potentially negative effect on cell health elicited by stress, and dysregulation of SG is implicated in many diseases.^120^^,^^121^ These non–membrane-bound organelles, which are mainly composed of proteins and RNAs, have liquidlike properties.^122^ They can fuse and flow like a liquid droplet, and molecules can undergo rapid exchange within the droplet as well as with their surrounding cytoplasm because of the lack of a physical barrier of lipid layers (which is commonly observed in membrane-bound organelles, such as mitochondria). Some of these non–membrane-bound organelles can mature into a gel-like state or solidify into amyloidlike aggregates.^123^ The formation of non–membrane-bound organelles is driven by the liquid-liquid phase separation from surrounding cytoplasm, leading to a condensed phase with participating molecules.^122^^,^^124^ The liquid-liquid phase separation is mediated by multivalent interactions from proteins and RNAs,^119^^,^^125^, ^126^, ^127^ among which the RNA-binding proteins with intrinsically disordered regions are overrepresented.^128^

**Figure 2:**
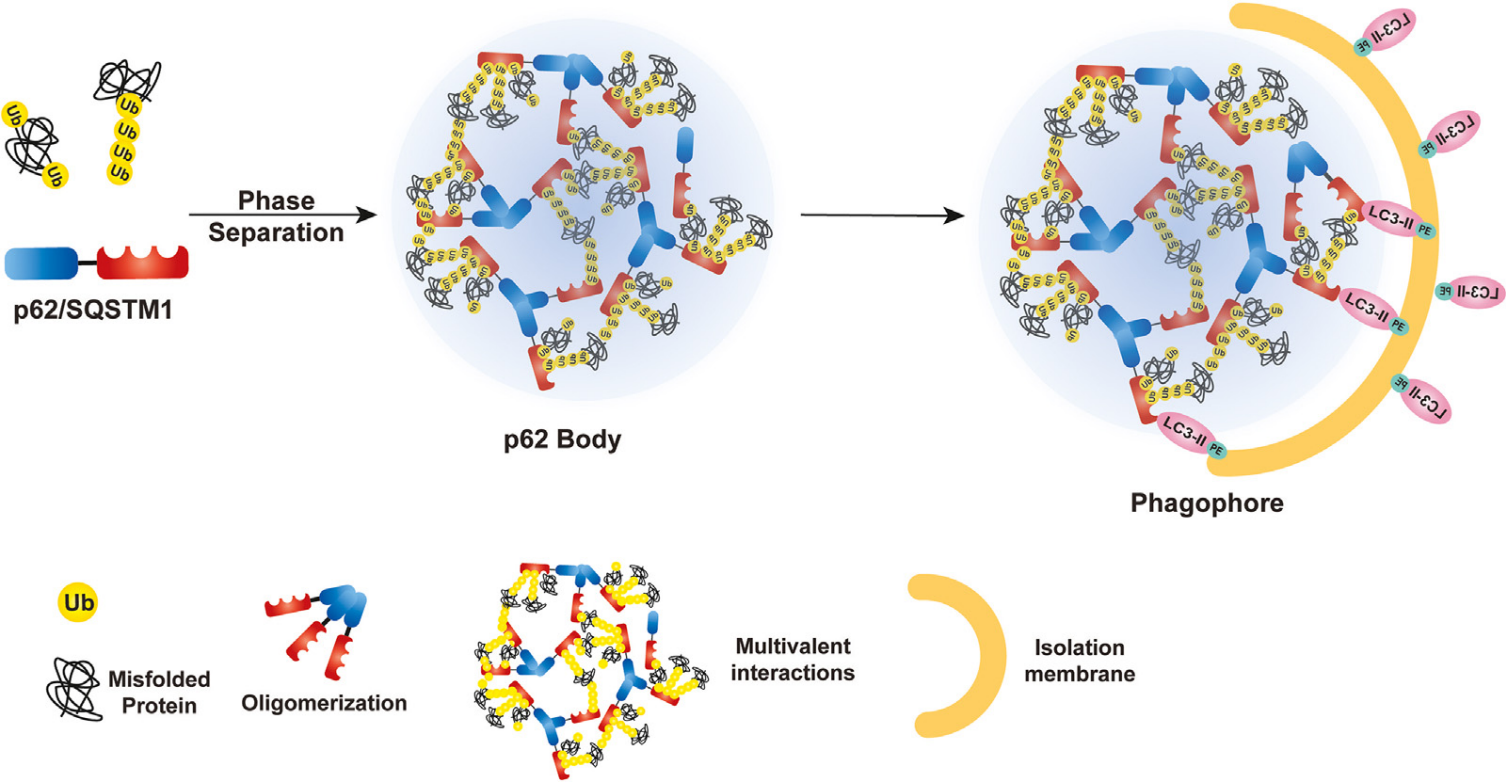
Formation and degradation of sequestosome 1 (SQSTM1/p62) bodies in selective autophagy. p62 forms homo- or hetero-oligomers via its Phox and Bem1 domain. Oligomerized p62 binds to polyubiquitinated substrates or ubiquitinated (Ub) misfolded proteins via its UB-associated domain, promoting the multivalent interactions to form larger membraneless p62 bodies. During selective autophagy process, p62 in p62 bodies can further tether with microtubule light chain 3 (LC3) via its LC3-interacting region domain to promote the degradation of p62 bodies via autophagic clearance. PE, phosphatidylethanolamine.

As a multivalent protein, p62 undergoes liquid–liquid-phase separation.^129^ p62 oligomerizes via its PB1 domain and thus clusters UBA domains from each p62 in the oligomers, creating a multivalent hub that is accessible to multiple partners.^115^^,^^129^^,^^130^ Overexpression of NBR1 blocks selective degradation of p62 and promotes the accumulation of phosphorylated p62 in liquidlike bodies.^72^ p62 droplets formed *in vivo* also show liquidlike properties, such as high sphericity, the ability to undergo fusion, and recovery after photobleaching.^131^ Liquidlike properties of the condensates are crucial in the initiation of a selective form of autophagy called aggrephagy.^115^^,^^129^^,^^130^ p62 acts as an RNA-binding protein, and p62 binds with vault RNAs, which are approximately 88- to 100-nt long noncoding RNAs, via the ZZ domain, and impairs selective autophagy for ubiquitinated proteins.^132^ Subsequent interactome studies show that p62 is enriched with recombinant signal binding proteins.^132^ Among these, seven of the 46 candidate p62 interactors are known as SG components^133^^,^^134^ In this way, p62 might also be involved in SG formation. SGs play a protective role in cells during the initial insult from the environment.^135^ However, an increasing body of evidence suggests that prolonged stress can cause the vitrification of SGs and turn into solid aggregates, which eventually develop into cytosolic inclusion bodies.^136^ Those inclusion bodies are also characterized with ubiquitinated proteins and are abundant with p62^137^, ^138^, ^139^ (Figure 2). Whether and how alcohol would affect SGs in hepatocytes is not clear, but it is likely that alcohol consumption may increase hepatic SG formation in early ALD, which may further progress to MDBs in patients with severe AH. Future studies are needed to further dissect the role of p62 in regulating intracellular SG formation and homeostasis in the pathogenesis of ALD.

## Role of Aggrephagy in the Clearance of Hepatic Protein Aggregates

As mentioned above, the formation of MDB and IHB inclusions caused by the aggregation of misfolded proteins is a hallmark of ALD. Soluble protein aggregates, such as a dimer, oligomer, or fibril, can be cytotoxic in the disease state due to increased proteotoxicity, whereas the formation of insoluble protein aggregates or inclusion is protective due to decreased proteotoxicity.^102^^,^^138^ There are two major protein degradation pathways in eukaryotic cells: the ubiquitin-proteasome system (UPS) and autophagy.^4^^,^^102^^,^^138^ Misfolded proteins typically refold with the help of chaperons or get degraded by UPS. However, when chaperons and UPS fail to act, these misfolded proteins tend to accumulate into protein aggregates and are removed by aggrephagy.^140^^,^^141^ Ubiquitination of misfolded proteins serves as a key signal for aggrephagy,^140^ where the ubiquitinated aggregates can be recognized by various autophagy receptors for degradation.^129^ Various autophagy cargo receptor proteins have been reported in aggrephagy, and they all contain LIR domain or putative LC3-interacting regions to interact with LC3 and drive misfolded proteins or aggregates for aggrephagy.^142^ These autophagy cargo receptor proteins include p62, NBR1, OPTN, nuclear dot protein 52, Toll-interacting protein, and tax1 binding protein 1 (a homology to nuclear dot protein 52).^140^^,^^142^, ^143^, ^144^ The eukaryotic chaperonin TCP-1 ring complex subunit chaperonin-containing TCP-1 subunit 2 acts as a chaperone and an aggrephagy receptor, which regulates aggrephagy by promoting autophagosome incorporation and clearance of protein aggregates via interacting with LC3 and ubiquitinated proteins.^145^

Ablations of essential autophagy-related genes, *Atg5* or *Atg7*, lead to the accumulation of ubiquitinated cytosolic protein aggregates in mice.^138^ Inhibition of both autophagy and the UPS promotes the accumulation of intracellular protein aggregates *in vitro*.^103^ However, pharmacologic inhibition of UPS activates autophagy to ameliorate the proteotoxicity by enhancing degradation of protein aggregates as an adaptive response.^102^^,^^103^^,^^146^ Pharmacologic activation of autophagy or genetic up-regulation of autophagy-related genes mitigate protein aggregate–induced cytotoxicity.^147^ In this setting, p62 connects the autophagy pathway and the UPS by promoting degradation of ubiquitinated protein aggregates and the formation of p62-positive protein aggregates.^138^^,^^148^ Notably, TRIM44 (tripartite motif containing 44) involves both the UPS system and aggrephagy by bridging the UPS and autophagy pathways. Decreased UPS activity leads to the up-regulation of TRIM44 that promotes ubiquitination and p62 oligomerization, which switch the degradation of protein aggregates via aggrephagy.^149^ There is a close interconnection between UPS and autophagy, and inhibition of UPS can trigger autophagy as an adaptive response to help regain proteostasis in cells.

As mentioned above, p62 is one of the major components of MDB and IHB. Genetic deletion of p62 failed to form the large MDB in mouse livers.^64^^,^^65^ In addition, the contents of p62 bodies, including misfolded protein and protein aggregates, are degraded by autophagy, which depends on a direct interaction of p62 oligomerization and its interaction with NBR1 via the PB1 domain, as well as interaction with LC3 via the LIR domain to initiate the phagophore formation.^129^^,^^131^^,^^150^ In addition to facilitating autophagic degradation of protein aggregates, p62 may also be involved in alcohol-induced formation of insoluble protein aggregates or condensates in young but not old mice.^151^ Nonmembrane p62 bodies could be both insoluble aggresome-like structures and low-liquidity gel-like p62 bodies.^131^^,^^138^^,^^152^ Polyubiquitin chains induce p62 phase separation to form gel-like p62 bodies *in vitro*, and gel-like p62 bodies cannot be taken up by autophagosomes in autophagy-defective *Atg12* knockout cells.^131^ Besides ubiquitinated protein aggregates, KEAP1 can also be sequestered in gel-like p62 bodies in mouse livers with impaired of LC3 interaction–dependent autophagy, resulting in persisting activation of NRF2 through the noncanonical NRF2 pathway.^152^ Nevertheless, the transition of gel-like p62 bodies to solid p62-positive aggregates (MDBs and IHBs) in the pathogenesis of ALD has not been studied. More studies are needed to further elucidate the role and mechanisms of turnover of the inclusions (especially p62 bodies) by aggrephagy in ALD.

## Conclusion and Future Perspectives

In the past decade, tremendous advances have been made in understanding the mechanisms and role of autophagy in liver pathophysiology and diseases such as ALD. Hepatocytes can use different types of selective autophagy as an adaptive response against various stress conditions in the liver. Pharmacologic activation of autophagy shows beneficial effects in various liver diseases, including ALD, in experimental animal models. However, some challenging questions remain to be answered. For instance, how p62 and other autophagy receptor proteins, separately or together, regulate different types of selective autophagy in complex liver disease conditions, such as ALD, is unclear. In addition to hepatocytes, alcohol affects almost all cell types, including hepatic stellate cells, cholangiocytes, endothelial cells, and Kupffer cells. It will be interesting to determine how p62 and autophagy in different cell types contribute to ALD. AH is characterized with excessive liver cell remodeling with increased hepatocyte degeneration and accumulation of fetal-like hepatic progenitor/ductular cells, resulting in liver failure. It remains to be determined whether manipulation of autophagy in different cell types would recover the hepatocyte identity from the de-differentiated hepatocytes and promote liver regeneration. Despite the great challenges, a better understanding of the autophagy receptor proteins and selective autophagy is expected to help yield promising therapeutic interventions to improve liver diseases such as ALD by precisely modulating specific selective autophagy.

## References

[1] Paik J.M., Golabi P., Younossi Y., Mishra A., Younossi Z.M. (2020). Changes in the global burden of chronic liver diseases from 2012 to 2017: the growing impact of NAFLD. Hepatology.

[2] World Health Organization (2019).

[3] Bruha R., Dvorak K., Petrtyl J. (2012). Alcoholic liver disease. World J Hepatol.

[4] Qian H., Chao X., Williams J., Fulte S., Li T., Yang L., Ding W.-X. (2021). Autophagy in liver diseases: a review. Mol Aspects Med.

[5] European Association for the Study of Liver (2012). EASL clinical practical guidelines: management of alcoholic liver disease. J Hepatol.

[6] Orman E.S., Odena G., Bataller R. (2013). Alcoholic liver disease: pathogenesis, management, and novel targets for therapy. J Gastroenterol Hepatol.

[7] Yeo Y.H., Zou B., Cheung R., Nguyen M.H. (2022). Increased mortality of patients with alcohol-related liver diseases during the COVID-19 pandemic in the United States. J Intern Med.

[8] Tikhanovich I., Kuravi S., Campbell R.V., Kharbanda K.K., Artigues A., Villar M.T., Weinman S.A. (2014). Regulation of FOXO3 by phosphorylation and methylation in hepatitis C virus infection and alcohol exposure. Hepatology.

[9] Dolganiuc A. (2015). Alcohol and viral hepatitis: role of lipid rafts. Alcohol Res.

[10] Julien J., Ayer T., Bethea E.D., Tapper E.B., Chhatwal J. (2020). Projected prevalence and mortality associated with alcohol-related liver disease in the USA, 2019–40: a modelling study. Lancet Public Health.

[11] World Health Organization (2018).

[12] Bataller R., Gao B. (2015). Liver fibrosis in alcoholic liver disease. Semin Liver Dis.

[13] World Health Organization (2020).

[14] Hayflick L. (2000). The future of ageing. Nature.

[15] Blazer D.G., Wu L.-T. (2009). The epidemiology of at-risk and binge drinking among middle-aged and elderly community adults: national survey on drug use and health. Am J Psychiatry.

[16] Seitz H.K., Stickel F. (2007). Alcoholic liver disease in the elderly. Clin Geriatr Med.

[17] Kim H., Kisseleva T., Brenner D.A. (2015). Aging and liver disease. Curr Opin Gastroenterol.

[18] Ren R., He Y., Ding D., Cui A., Bao H., Ma J., Hou X., Li Y., Feng D., Li X., Liangpunsakul S., Gao B., Wang H. (2022). Aging exaggerates acute-on-chronic alcohol-induced liver injury in mice and humans by inhibiting neutrophilic sirtuin 1-C/EBPalpha-miRNA-223 axis. Hepatology.

[19] Meier P., Seitz H.K. (2008). Age, alcohol metabolism and liver disease. Curr Opin Clin Nutr Metab Care.

[20] Timchenko N.A. (2009). Aging and liver regeneration. Trends Endocrinol Metab.

[21] Cursio R., Colosetti P., Codogno P., Cuervo A.M., Shen H.M. (2015). The role of autophagy in liver diseases: mechanisms and potential therapeutic targets. Biomed Res Int.

[22] Fernando R., Castro J.P., Flore T., Deubel S., Grune T., Ott C. (2020). Age-related maintenance of the autophagy-lysosomal system is dependent on skeletal muscle type. Oxid Med Cell Longev.

[23] Liang W.J., Moyzis A.G., Lampert M.A., Diao R.C.Y., Najor R.H., Gustafsson A.B. (2020). Aging is associated with a decline in Atg9b-mediated autophagosome formation and appearance of enlarged mitochondria in the heart. Aging Cell.

[24] Prado V., Caballería J., Vargas V., Bataller R., Altamirano J. (2016). Alcoholic hepatitis: how far are we and where are we going?. Ann Hepatol.

[25] Louvet A., Mathurin P. (2015). Alcoholic liver disease: mechanisms of injury and targeted treatment. Nat Rev Gastroenterol Hepatol.

[26] Parzych K.R., Klionsky D.J. (2014). An overview of autophagy: morphology, mechanism, and regulation. Antioxid Redox Signal.

[27] Yan S., Khambu B., Hong H., Liu G., Huda N., Yin X.-M. (2019). Autophagy, metabolism, and alcohol-related liver disease: novel modulators and functions. Int J Mol Sci.

[28] Cuervo A.M., Dice J.F. (1996). A receptor for the selective uptake and degradation of proteins by lysosomes. Science.

[29] Schneider J.L., Villarroya J., Diaz-Carretero A., Patel B., Urbanska A.M., Thi M.M., Villarroya F., Santambrogio L., Cuervo A.M. (2015). Loss of hepatic chaperone-mediated autophagy accelerates proteostasis failure in aging. Aging Cell.

[30] Yin X.M., Ding W.X., Gao W. (2008). Autophagy in the liver. Hepatology.

[31] Rautou P.E., Mansouri A., Lebrec D., Durand F., Valla D., Moreau R. (2010). Autophagy in liver diseases. J Hepatol.

[32] Ding W.X. (2010). Role of autophagy in liver physiology and pathophysiology. World J Biol Chem.

[33] Toshima T., Shirabe K., Fukuhara T., Ikegami T., Yoshizumi T., Soejima Y., Ikeda T., Okano S., Maehara Y. (2014). Suppression of autophagy during liver regeneration impairs energy charge and hepatocyte senescence in mice. Hepatology.

[34] Chao X., Ding W.X. (2019). Role and mechanisms of autophagy in alcohol-induced liver injury. Adv Pharmacol.

[35] Chao X., Wang S., Zhao K., Li Y., Williams J.A., Li T., Chavan H., Krishnamurthy P., He X.C., Li L., Ballabio A., Ni H.M., Ding W.X. (2018). Impaired TFEB-mediated lysosome biogenesis and autophagy promote chronic ethanol-induced liver injury and steatosis in mice. Gastroenterology.

[36] Chao X., Williams S.N., Ding W.X. (2022). Role of mechanistic target of rapamycin in autophagy and alcohol-associated liver disease. Am J Physiol Cell Physiol.

[37] Chao X., Qian H., Wang S., Fulte S., Ding W.-X. (2020). Autophagy and liver cancer. Clin Mol Hepatol.

[38] Takamura A., Komatsu M., Hara T., Sakamoto A., Kishi C., Waguri S., Eishi Y., Hino O., Tanaka K., Mizushima N. (2011). Autophagy-deficient mice develop multiple liver tumors. Genes Dev.

[39] Ni H.M., Woolbright B.L., Williams J., Copple B., Cui W., Luyendyk J.P., Jaeschke H., Ding W.X. (2014). Nrf2 promotes the development of fibrosis and tumorigenesis in mice with defective hepatic autophagy. J Hepatol.

[40] Chao X., Wang S., Fulte S., Ma X., Ahamed F., Cui W., Liu Z., Rulicke T., Zatloukal K., Zong W.X., Liu W., Ni H.M., Ding W.X. (2022). Hepatocytic p62 suppresses ductular reaction and tumorigenesis in mouse livers with mTORC1 activation and defective autophagy. J Hepatol.

[41] Ni H.M., Chao X., Yang H., Deng F., Wang S., Bai Q., Qian H., Cui Y., Cui W., Shi Y., Zong W.X., Wang Z., Yang L., Ding W.X. (2019). Dual roles of mammalian target of rapamycin in regulating liver injury and tumorigenesis in autophagy-defective mouse liver. Hepatology.

[42] Huda N., Khambu B., Liu G., Nakatsumi H., Yan S.M., Chen X.Y., Ma M.C.L., Dong Z., Nakayama K.I., Yin X.M. (2022). Senescence connects autophagy deficiency to inflammation and tumor progression in the liver. Cell Mol Gastroenterol Hepatol.

[43] Liu P.F., Anandhan A., Chen J.J., Shakya A., Dodson M., Ooi A., Chapman E., White E., Garcia J.G.N., Zhang D.D. (2023). Decreased autophagosome biogenesis, reduced NRF2, and enhanced ferroptotic cell death are underlying molecular mechanisms of non-alcoholic fatty liver disease. Redox Biol.

[44] Ding W.X., Li M., Chen X., Ni H.M., Lin C.W., Gao W., Lu B., Stolz D.B., Clemens D.L., Yin X.M. (2010). Autophagy reduces acute ethanol-induced hepatotoxicity and steatosis in mice. Gastroenterology.

[45] Chao X., Wang S., Zhao K., Li Y., Williams J.A., Li T., Chavan H., Krishnamurthy P., He X.C., Li L. (2018). Impaired TFEB-mediated lysosome biogenesis and autophagy promote chronic ethanol-induced liver injury and steatosis in mice. Gastroenterology.

[46] Ma X., Chen A., Melo L., Clemente-Sanchez A., Chao X., Ahmadi A.R., Peiffer B., Sun Z., Sesaki H., Li T. (2023). Loss of hepatic DRP1 exacerbates alcoholic hepatitis by inducing megamitochondria and mitochondrial maladaptation. Hepatology.

[47] Williams J.A., Ding W.-X. (2020). Role of autophagy in alcohol and drug-induced liver injury. Food Chem Toxicol.

[48] Rasineni K., Donohue T.M., Thomes P.G., Yang L., Tuma D.J., McNiven M.A., Casey C.A. (2017). Ethanol-induced steatosis involves impairment of lipophagy, associated with reduced Dynamin2 activity. Hepatol Commun.

[49] Schulze R.J., Rasineni K., Weller S.G., Schott M.B., Schroeder B., Casey C.A., McNiven M.A. (2017). Ethanol exposure inhibits hepatocyte lipophagy by inactivating the small guanosine triphosphatase Rab7. Hepatol Commun.

[50] Li Y., Ding W.X. (2017). Impaired Rab7 and dynamin2 block fat turnover by autophagy in alcoholic fatty livers. Hepatol Commun.

[51] Wang L., Zhou J., Yan S., Lei G., Lee C.-H., Yin X.-M. (2017). Ethanol-triggered lipophagy requires SQSTM1 in AML12 hepatic cells. Sci Rep.

[52] Zeng H., Guo X., Zhou F., Xiao L., Liu J., Jiang C., Xing M., Yao P. (2019). Quercetin alleviates ethanol-induced liver steatosis associated with improvement of lipophagy. Food Chem Toxicol.

[53] Li Y., Chao X., Yang L., Lu Q., Li T., Ding W.X., Ni H.M. (2018). Impaired fasting-induced adaptive lipid droplet biogenesis in liver-specific Atg5-deficient mouse liver is mediated by persistent nuclear factor-like 2 activation. Am J Pathol.

[54] Takahashi S.S., Sou Y.S., Saito T., Kuma A., Yabe T., Sugiura Y., Lee H.C., Suematsu M., Yokomizo T., Koike M., Terai S., Mizushima N., Waguri S., Komatsu M. (2020). Loss of autophagy impairs physiological steatosis by accumulation of NCoR1. Life Sci Alliance.

[55] Ma D., Molusky M.M., Song J., Hu C.R., Fang F., Rui C., Mathew A.V., Pennathur S., Liu F., Cheng J.X., Guan J.L., Lin J.D. (2013). Autophagy deficiency by hepatic FIP200 deletion uncouples steatosis from liver injury in NAFLD. Mol Endocrinol.

[56] Ding W.X., Ni H.M., Waguri S., Komatsu M. (2022). Lack of hepatic autophagy promotes severity of liver injury but not steatosis. J Hepatol.

[57] Ding W.-X., Li M., Yin X.-M. (2011). Selective taste of ethanol-induced autophagy for mitochondria and lipid droplets. Autophagy.

[58] García-Ruiz C., Kaplowitz N., Fernandez-Checa J.C. (2013). Role of mitochondria in alcoholic liver disease. Curr Pathobiol Rep.

[59] Yu X., Xu Y., Zhang S., Sun J., Liu P., Xiao L., Tang Y., Liu L., Yao P. (2016). Quercetin attenuates chronic ethanol-induced hepatic mitochondrial damage through enhanced mitophagy. Nutrients.

[60] Williams J.A., Ding W.X. (2015). A mechanistic review of mitophagy and its role in protection against alcoholic liver disease. Biomolecules.

[61] Ma X., Chen A., Melo L., Clemente-Sanchez A., Chao X., Ahmadi A.R., Peiffer B., Sun Z., Sesaki H., Li T., Wang X., Liu W., Bataller R., Ni H.M., Ding W.X. (2023). Loss of hepatic DRP1 exacerbates alcoholic hepatitis by inducing megamitochondria and mitochondrial maladaptation. Hepatology.

[62] Williams J.A., Ni H.M., Ding Y., Ding W.X. (2015). Parkin regulates mitophagy and mitochondrial function to protect against alcohol-induced liver injury and steatosis in mice. Am J Physiol Gastrointest Liver Physiol.

[63] Eid N., Ito Y., Horibe A., Otsuki Y. (2016). Ethanol-induced mitophagy in liver is associated with activation of the PINK1-Parkin pathway triggered by oxidative DNA damage. Histol Histopathol.

[64] Zatloukal K., French S.W., Stumptner C., Strnad P., Harada M., Toivola D.M., Cadrin M., Omary M.B. (2007). From Mallory to Mallory-denk bodies: what, how and why?. Exp Cell Res.

[65] Lahiri P., Schmidt V., Smole C., Kufferath I., Denk H., Strnad P., Rulicke T., Frohlich L.F., Zatloukal K. (2016). p62/Sequestosome-1 is indispensable for maturation and stabilization of mallory-denk bodies. PLoS One.

[66] Komatsu M., Kurokawa H., Waguri S., Taguchi K., Kobayashi A., Ichimura Y., Sou Y.S., Ueno I., Sakamoto A., Tong K.I., Kim M., Nishito Y., Iemura S., Natsume T., Ueno T., Kominami E., Motohashi H., Tanaka K., Yamamoto M. (2010). The selective autophagy substrate p62 activates the stress responsive transcription factor Nrf2 through inactivation of Keap1. Nat Cell Biol.

[67] Ni H.M., Boggess N., McGill M.R., Lebofsky M., Borude P., Apte U., Jaeschke H., Ding W.X. (2012). Liver-specific loss of Atg5 causes persistent activation of Nrf2 and protects against acetaminophen-induced liver injury. Toxicol Sci.

[68] Katsuragi Y., Ichimura Y., Komatsu M. (2015). p62/SQSTM 1 functions as a signaling hub and an autophagy adaptor. FEBS J.

[69] Komatsu M., Ichimura Y. (2010). Physiological significance of selective degradation of p62 by autophagy. FEBS Lett.

[70] Manley S., Williams J.A., Ding W.-X. (2013). Role of p62/SQSTM1 in liver physiology and pathogenesis. Exp Biol Med.

[71] Ren J., Wang J., Wang Z., Wu J. (2014). Structural and biochemical insights into the homotypic PB1-PB1 complex between PKCζ and p62. Sci China Life Sci.

[72] Sánchez-Martín P., Sou Y.S., Kageyama S., Koike M., Waguri S., Komatsu M. (2020). NBR 1-mediated p62-liquid droplets enhance the Keap1-Nrf2 system. EMBO Rep.

[73] Nakamura K., Kimple A.J., Siderovski D.P., Johnson G.L. (2010). PB1 domain interaction of p62/sequestosome 1 and MEKK3 regulates NF-κB activation. J Biol Chem.

[74] Zhang Y., Mun S.R., Linares J.F., Ahn J., Towers C.G., Ji C.H., Fitzwalter B.E., Holden M.R., Mi W., Shi X. (2018). ZZ-dependent regulation of p62/SQSTM1 in autophagy. Nat Commun.

[75] Duran A., Amanchy R., Linares J.F., Joshi J., Abu-Baker S., Porollo A., Hansen M., Moscat J., Diaz-Meco M.T. (2011). p62 is a key regulator of nutrient sensing in the mTORC1 pathway. Mol Cell.

[76] Ichimura Y., Kumanomidou T., Sou Y.S., Mizushima T., Ezaki J., Ueno T., Kominami E., Yamane T., Tanaka K., Komatsu M. (2008). Structural basis for sorting mechanism of p62 in selective autophagy. J Biol Chem.

[77] Pankiv S., Clausen T.H., Lamark T., Brech A., Bruun J.-A., Outzen H., Øvervatn A., Bjørkøy G., Johansen T. (2007). p62/SQSTM1 binds directly to Atg8/LC3 to facilitate degradation of ubiquitinated protein aggregates by autophagy. J Biol Chem.

[78] Katsuragi Y., Ichimura Y., Komatsu M. (2016). Regulation of the Keap1–Nrf2 pathway by p62/SQSTM1. Curr Opin Toxicol.

[79] Ichimura Y., Waguri S., Sou Y.S., Kageyama S., Hasegawa J., Ishimura R., Saito T., Yang Y., Kouno T., Fukutomi T., Hoshii T., Hirao A., Takagi K., Mizushima T., Motohashi H., Lee M.S., Yoshimori T., Tanaka K., Yamamoto M., Komatsu M. (2013). Phosphorylation of p62 activates the Keap1-Nrf2 pathway during selective autophagy. Mol Cell.

[80] Jain A., Lamark T., Sjottem E., Larsen K.B., Awuh J.A., Overvatn A., McMahon M., Hayes J.D., Johansen T. (2010). p62/SQSTM1 is a target gene for transcription factor NRF2 and creates a positive feedback loop by inducing antioxidant response element-driven gene transcription. J Biol Chem.

[81] Inami Y., Waguri S., Sakamoto A., Kouno T., Nakada K., Hino O., Watanabe S., Ando J., Iwadate M., Yamamoto M. (2011). Persistent activation of Nrf2 through p62 in hepatocellular carcinoma cells. J Cell Biol.

[82] Isogai S., Morimoto D., Arita K., Unzai S., Tenno T., Hasegawa J., Sou Y.S., Komatsu M., Tanaka K., Shirakawa M., Tochio H. (2011). Crystal structure of the ubiquitin-associated (UBA) domain of p62 and its interaction with ubiquitin. J Biol Chem.

[83] Wurzer B., Zaffagnini G., Fracchiolla D., Turco E., Abert C., Romanov J., Martens S. (2015). Oligomerization of p62 allows for selection of ubiquitinated cargo and isolation membrane during selective autophagy. Elife.

[84] Matsumoto G., Wada K., Okuno M., Kurosawa M., Nukina N. (2011). Serine 403 phosphorylation of p62/SQSTM1 regulates selective autophagic clearance of ubiquitinated proteins. Mol Cell.

[85] Matsumoto G., Shimogori T., Hattori N., Nukina N. (2015). TBK1 controls autophagosomal engulfment of polyubiquitinated mitochondria through p62/SQSTM1 phosphorylation. Hum Mol Genet.

[86] Lau A., Wang X.-J., Zhao F., Villeneuve N.F., Wu T., Jiang T., Sun Z., White E., Zhang D.D. (2010). A noncanonical mechanism of Nrf2 activation by autophagy deficiency: direct interaction between Keap1 and p62. Mol Cell Biol.

[87] Jena K.K., Kolapalli S.P., Mehto S., Nath P., Das B., Sahoo P.K., Ahad A., Syed G.H., Raghav S.K., Senapati S. (2018). TRIM16 controls assembly and degradation of protein aggregates by modulating the p62-NRF2 axis and autophagy. EMBO J.

[88] Pan J.-A., Sun Y., Jiang Y.-P., Bott A.J., Jaber N., Dou Z., Yang B., Chen J.-S., Catanzaro J.M., Du C. (2016). TRIM21 ubiquitylates SQSTM1/p62 and suppresses protein sequestration to regulate redox homeostasis. Mol Cell.

[89] Wang F., Zhang Y., Shen J., Yang B., Dai W., Yan J., Maimouni S., Daguplo H.Q., Coppola S., Gao Y., Wang Y., Du Z., Peng K., Liu H., Zhang Q., Tang F., Wang P., Gao S., Wang Y., Ding W.X., Guo G., Wang F., Zong W.X. (2021). The ubiquitin E3 ligase TRIM21 promotes hepatocarcinogenesis by suppressing the p62-Keap1-Nrf2 antioxidant pathway. Cell Mol Gastroenterol Hepatol.

[90] Moscat J., Diaz-Meco M.T. (2009). p62 at the crossroads of autophagy, apoptosis, and cancer. Cell.

[91] Sanchez-Martin P., Komatsu M. (2018). p62/SQSTM1 - steering the cell through health and disease. J Cell Sci.

[92] You Z.Y., Jiang W.X., Qin L.Y., Gong Z., Wan W., Li J., Wang Y.S., Zhang H.T., Peng C., Zhou T.H., Tang C., Liu W. (2019). Requirement for p62 acetylation in the aggregation of ubiquitylated proteins under nutrient stress. Nat Commun.

[93] Kirkin V., Lamark T., Sou Y.-S., Bjørkøy G., Nunn J.L., Bruun J.-A., Shvets E., McEwan D.G., Clausen T.H., Wild P. (2009). A role for NBR1 in autophagosomal degradation of ubiquitinated substrates. Mol Cell.

[94] Sanz L., Sanchez P., Lallena M.-J., Diaz-Meco M.T., Moscat J. (1999). The interaction of p62 with RIP links the atypical PKCs to NF-κB activation. EMBO J.

[95] Sanz L., Diaz-Meco M.T., Nakano H., Moscat J. (2000). The atypical PKC-interacting protein p62 channels NF-κB activation by the IL-1–TRAF6 pathway. EMBO J.

[96] Wooten M.W., Geetha T., Seibenhener M.L., Babu J.R., Diaz-Meco M.T., Moscat J. (2005). The p62 scaffold regulates nerve growth factor-induced NF-κB activation by influencing TRAF6 polyubiquitination. J Biol Chem.

[97] Durán A., Serrano M., Leitges M., Flores J.M., Picard S., Brown J.P., Moscat J., Diaz-Meco M.T. (2004). The atypical PKC-interacting protein p62 is an important mediator of RANK-activated osteoclastogenesis. Dev Cell.

[98] Schimmack G., Schorpp K., Kutzner K., Gehring T., Brenke J.K., Hadian K., Krappmann D. (2017). YOD1/TRAF6 association balances p62-dependent IL-1 signaling to NF-κB. Elife.

[99] Denk H., Stumptner C., Fuchsbichler A., Müller T., Farr G., Müller W., Terracciano L., Zatloukal K. (2006). Are the mallory bodies and intracellular hyaline bodies in neoplastic and non-neoplastic hepatocytes related?. J Pathol A J Pathological Soc Great Britain Ireland.

[100] Chelliah A.R., Radhi J.M. (2016). Hepatocellular carcinoma with prominent intracytoplasmic inclusions: a report of two cases. Case Rep Hepatol.

[101] Zatloukal K., Stumptner C., Fuchsbichler A., Heid H., Schnoelzer M., Kenner L., Kleinert R., Prinz M., Aguzzi A., Denk H. (2002). p62 Is a common component of cytoplasmic inclusions in protein aggregation diseases. Am J Pathol.

[102] Ding W.X., Yin X.M. (2008). Sorting, recognition and activation of the misfolded protein degradation pathways through macroautophagy and the proteasome. Autophagy.

[103] Ding W.X., Ni H.M., Gao W., Yoshimori T., Stolz D.B., Ron D., Yin X.M. (2007). Linking of autophagy to ubiquitin-proteasome system is important for the regulation of endoplasmic reticulum stress and cell viability. Am J Pathol.

[104] French S.W., Nash J., Shitabata P., Kachi K., Hara C., Chedid A., Mendenhall C.L. (1993). Pathology of alcoholic liver disease: VA Cooperative Study Group 119. Semin Liver Dis.

[105] Torruellas C., French S.W., Medici V. (2014). Diagnosis of alcoholic liver disease. World J Gastroenterol.

[106] Mendler M.H., Kanel G., Govindarajan S. (2005). Proposal for a histological scoring and grading system for non-alcoholic fatty liver disease. Liver Int.

[107] Kucukoglu O., Guldiken N., Chen Y., Usachov V., El-Heliebi A., Haybaeck J., Denk H., Trautwein C., Strnad P. (2014). High-fat diet triggers Mallory-Denk body formation through misfolding and crosslinking of excess keratin 8. Hepatology.

[108] Hanada S., Snider N.T., Brunt E.M., Hollenberg P.F., Omary M.B. (2010). Gender dimorphic formation of mouse Mallory-Denk bodies and the role of xenobiotic metabolism and oxidative stress. Gastroenterology.

[109] Hanada S., Harada M., Abe M., Akiba J., Sakata M., Kwan R., Taniguchi E., Kawaguchi T., Koga H., Nagata E. (2012). Aging modulates susceptibility to mouse liver Mallory-Denk body formation. J Histochem Cytochem.

[110] Nagy L.E., Ding W.X., Cresci G., Saikia P., Shah V.H. (2016). Linking pathogenic mechanisms of alcoholic liver disease with clinical phenotypes. Gastroenterology.

[111] Furuya S., Argemi J., Uehara T., Katou Y., Fouts D.E., Schnabl B., Dubuquoy L., Belorkar A., Vadigepalli R., Kono H., Bataller R., Rusyn I. (2020). A novel mouse model of acute-on-chronic cholestatic alcoholic liver disease: a systems biology comparison with human alcoholic hepatitis. Alcohol Clin Exp Res.

[112] Kwan R., Hanada S., Harada M., Strnad P., Li D.H., Omary M.B. (2012). Keratin 8 phosphorylation regulates its transamidation and hepatocyte Mallory-Denk body formation. FASEB J.

[113] Strnad P., Harada M., Siegel M., Terkeltaub R.A., Graham R.M., Khosla C., Omary M.B. (2007). Transglutaminase 2 regulates Mallory body inclusion formation and injury-associated liver enlargement. Gastroenterology.

[114] French S., Masouminia M., Samadzadeh S., Tillman B., Mendoza A., French B. (2017). Role of protein quality control failure in alcoholic hepatitis pathogenesis. Biomolecules.

[115] Cabe M., Rademacher D.J., Karlsson A.B., Cherukuri S., Bakowska J.C. (2018). PB1 and UBA domains of p62 are essential for aggresome-like induced structure formation. Biochem Biophys Res Commun.

[116] Aigelsreiter A., Neumann J., Pichler M., Halasz J., Zatloukal K., Berghold A., Douschan P., Rainer F., Stauber R., Haybaeck J. (2017). Hepatocellular carcinomas with intracellular hyaline bodies have a poor prognosis. Liver Int.

[117] Aishima S., Fujita N., Mano Y., Iguchi T., Taketomi A., Maehara Y., Oda Y., Tsuneyoshi M. (2010). p62+ Hyaline inclusions in intrahepatic cholangiocarcinoma associated with viral hepatitis or alcoholic liver disease. Am J Pathol.

[118] Komatsu M., Waguri S., Koike M., Sou Y-s, Ueno T., Hara T., Mizushima N., Iwata J-i, Ezaki J., Murata S. (2007). Homeostatic levels of p62 control cytoplasmic inclusion body formation in autophagy-deficient mice. Cell.

[119] Banani S.F., Lee H.O., Hyman A.A., Rosen M.K. (2017). Biomolecular condensates: organizers of cellular biochemistry. Nat Rev Mol Cell Biol.

[120] Protter D.S., Parker R. (2016). Principles and properties of stress granules. Trends Cell Biol.

[121] Wolozin B., Ivanov P. (2019). Stress granules and neurodegeneration. Nat Rev Neurosci.

[122] Hyman A.A., Weber C.A., Jülicher F. (2014). Liquid-liquid phase separation in biology. Annu Rev Cell Dev Biol.

[123] Boeynaems S., Alberti S., Fawzi N.L., Mittag T., Polymenidou M., Rousseau F., Schymkowitz J., Shorter J., Wolozin B., Van Den Bosch L. (2018). Protein phase separation: a new phase in cell biology. Trends Cell Biol.

[124] Alberti S. (2017). Phase separation in biology. Curr Biol.

[125] Posey A.E., Holehouse A.S., Pappu R.V. (2018). Phase separation of intrinsically disordered proteins. Methods Enzymol.

[126] Li P., Banjade S., Cheng H.-C., Kim S., Chen B., Guo L., Llaguno M., Hollingsworth J.V., King D.S., Banani S.F. (2012). Phase transitions in the assembly of multivalent signalling proteins. Nature.

[127] Harmon T.S., Holehouse A.S., Rosen M.K., Pappu R.V. (2017). Intrinsically disordered linkers determine the interplay between phase separation and gelation in multivalent proteins. Elife.

[128] Harrison A.F., Shorter J. (2017). RNA-binding proteins with prion-like domains in health and disease. Biochem J.

[129] Sun D., Wu R., Li P., Yu L. (2020). Phase separation in regulation of aggrephagy. J Mol Biol.

[130] Wilson M.I., Gill D.J., Perisic O., Quinn M.T., Williams R.L. (2003). PB1 domain-mediated heterodimerization in NADPH oxidase and signaling complexes of atypical protein kinase C with Par6 and p62. Mol Cell.

[131] Sun D., Wu R., Zheng J., Li P., Yu L. (2018). Polyubiquitin chain-induced p62 phase separation drives autophagic cargo segregation. Cell Res.

[132] Horos R., Buscher M., Kleinendorst R., Alleaume A.M., Tarafder A.K., Schwarzl T., Dziuba D., Tischer C., Zielonka E.M., Adak A., Castello A., Huber W., Sachse C., Hentze M.W. (2019). The small non-coding vault RNA1-1 acts as a riboregulator of autophagy. Cell.

[133] Chitiprolu M., Jagow C., Tremblay V., Bondy-Chorney E., Paris G., Savard A., Palidwor G., Barry F.A., Zinman L., Keith J. (2018). A complex of C9ORF72 and p62 uses arginine methylation to eliminate stress granules by autophagy. Nat Commun.

[134] Vanderweyde T., Youmans K., Liu-Yesucevitz L., Wolozin B. (2013). Role of stress granules and RNA-binding proteins in neurodegeneration: a mini-review. Gerontology.

[135] Barr J.E., Munyikwa M.R., Frazier E.A., Hinton S.D. (2013). The pseudophosphatase MK-STYX inhibits stress granule assembly independently of Ser149 phosphorylation of G3BP-1. FEBS J.

[136] Mateju D., Franzmann T.M., Patel A., Kopach A., Boczek E.E., Maharana S., Lee H.O., Carra S., Hyman A.A., Alberti S. (2017). An aberrant phase transition of stress granules triggered by misfolded protein and prevented by chaperone function. EMBO J.

[137] Anderson E.N., Gochenaur L., Singh A., Grant R., Patel K., Watkins S., Wu J.Y., Pandey U.B. (2018). Traumatic injury induces stress granule formation and enhances motor dysfunctions in ALS/FTD models. Hum Mol Genet.

[138] Tan S., Wong E. (2017). Kinetics of protein aggregates disposal by aggrephagy. Methods Enzymol.

[139] Ma S., Attarwala I., Xie X.-Q. (2019). SQSTM1/p62: a potential target for neurodegenerative disease. ACS Chem Neurosci.

[140] Lamark T., Johansen T. (2012). Aggrephagy: selective disposal of protein aggregates by macroautophagy. Int J Cell Biol.

[141] Dikic I. (2017). Proteasomal and autophagic degradation systems. Annu Rev Biochem.

[142] Tumbarello D.A., Manna P.T., Allen M., Bycroft M., Arden S.D., Kendrick-Jones J., Buss F. (2015). The autophagy receptor TAX1BP1 and the molecular motor myosin VI are required for clearance of salmonella typhimurium by autophagy. PLoS Pathog.

[143] Sarraf S.A., Shah H.V., Kanfer G., Ward M.E., Youle R.J. (2019). Selective autophagic clearance of protein aggregates is mediated by the autophagy receptor, Tax1bp1. bioRxiv.

[144] Lystad A.H., Simonsen A. (2015). Assays to monitor aggrephagy. Methods.

[145] Ma X., Lu C., Chen Y., Li S., Ma N., Tao X., Li Y., Wang J., Zhou M., Yan Y.-B. (2022). CCT2 is an aggrephagy receptor for clearance of solid protein aggregates. Cell.

[146] Zhu K., Dunner K., McConkey D.J. (2010). Proteasome inhibitors activate autophagy as a cytoprotective response in human prostate cancer cells. Oncogene.

[147] Pattison J.S., Osinska H., Robbins J. (2011). Atg7 induces basal autophagy and rescues autophagic deficiency in CryABR120G cardiomyocytes. Circ Res.

[148] Liu W.J., Ye L., Huang W.F., Guo L.J., Xu Z.G., Wu H.L., Yang C., Liu H.F. (2016). p62 links the autophagy pathway and the ubiqutin–proteasome system upon ubiquitinated protein degradation. Cell Mol Biol Lett.

[149] Lyu L., Chen Z., McCarty N. (2022). TRIM44 links the UPS to SQSTM1/p62-dependent aggrephagy and removing misfolded proteins. Autophagy.

[150] Danieli A., Martens S. (2018). p62-mediated phase separation at the intersection of the ubiquitin-proteasome system and autophagy. J Cell Sci.

[151] Qian H., Chao X., Wang S., Li Y., Jiang X., Sun Z., Rulicke T., Zatloukal K., Ni H.M., Ding W.X. (2023). Loss of SQSTM1/p62 induces obesity and exacerbates alcohol-induced liver injury in aged mice. Cell Mol Gastroenterol Hepatol.

[152] Kageyama S., Gudmundsson S.R., Sou Y.S., Ichimura Y., Tamura N., Kazuno S., Ueno T., Miura Y., Noshiro D., Abe M., Mizushima T., Miura N., Okuda S., Motohashi H., Lee J.A., Sakimura K., Ohe T., Noda N.N., Waguri S., Eskelinen E.L., Komatsu M. (2021). p62/SQSTM1-droplet serves as a platform for autophagosome formation and anti-oxidative stress response. Nat Commun.

